# Supplementary figures and images for: GobyWeb: Simplified Management and Analysis of Gene Expression and DNA Methylation Sequencing Data
Source: PLoS One. 2013 Jul 23;8(7):e69666. doi: 10.1371/journal.pone.0069666 (PMC3720652; doi:10.1371/journal.pone.0069666)

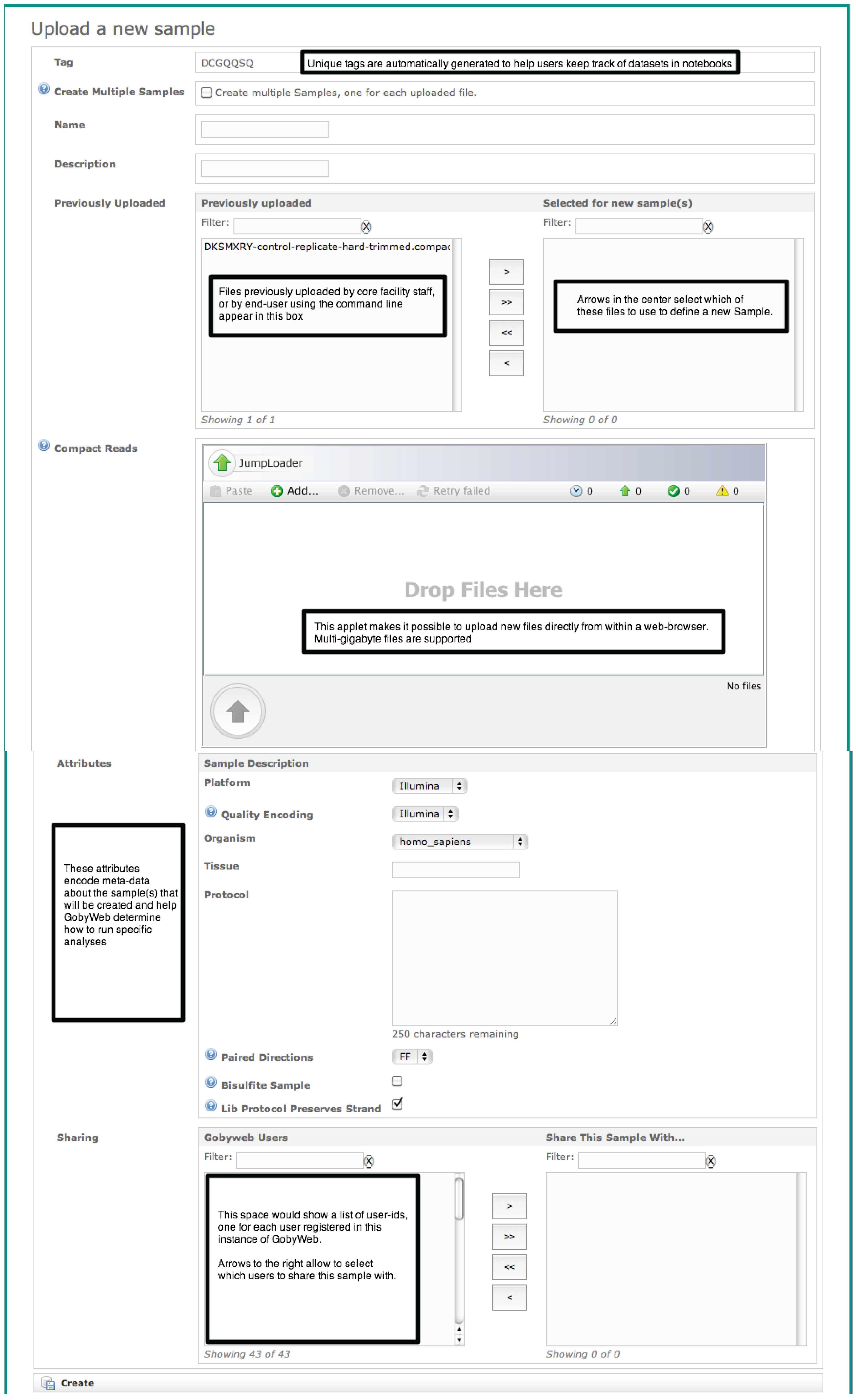

Supplement: Figure S1 — Uploading reads into GobyWeb to create a new Sample. Read files can be uploaded in a variety of file formats. When the checkbox “Create Multiple Samples” is not selected, individual files are concatenated to yield a single independent biological sample. When the box is not checked, multiple samples are created and associated with the meta-data described on the form. (TIFF) [file pone.0069666.s001.tiff]

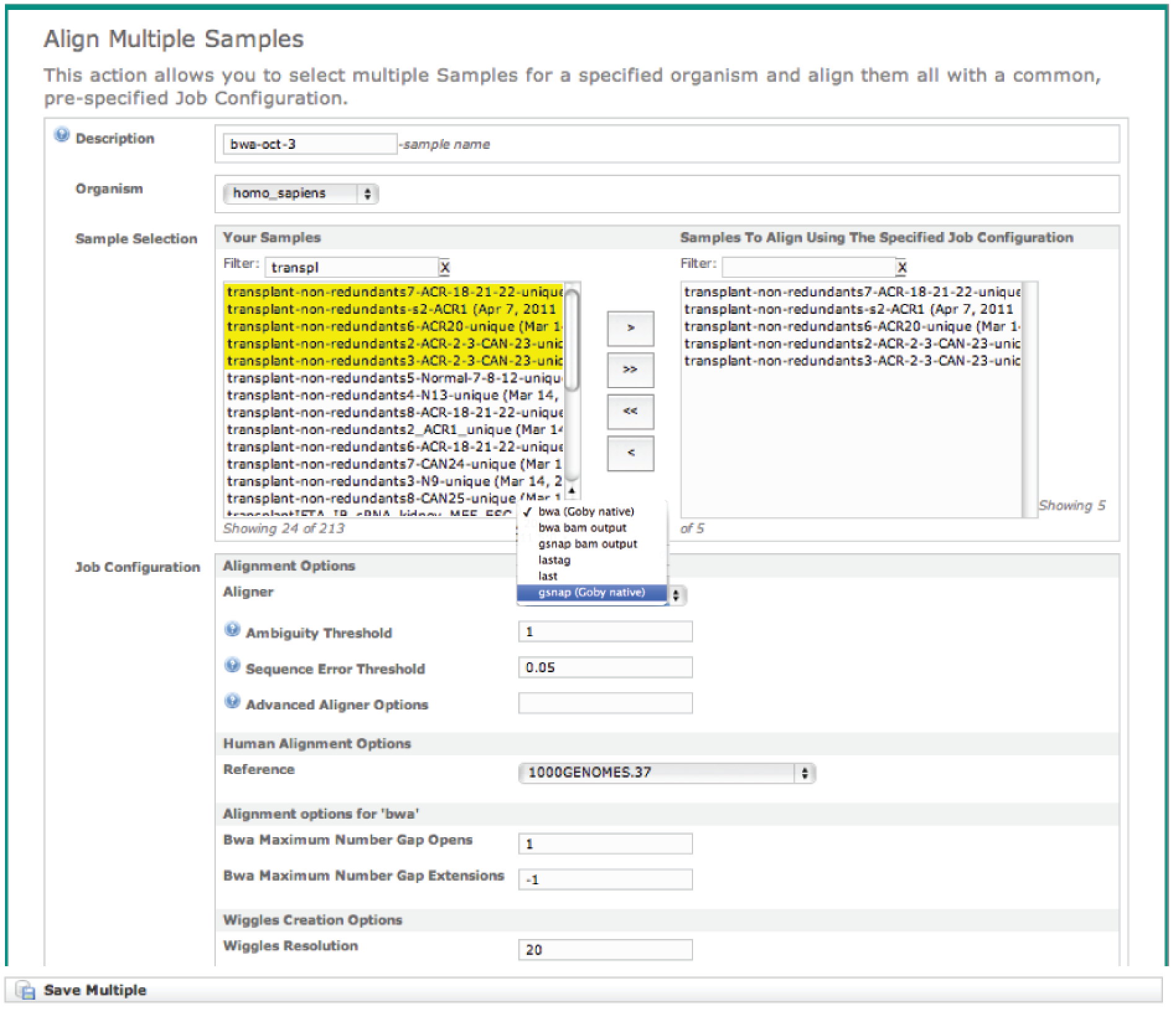

Supplement: Figure S2 — Consistent alignment of multiple samples. GobyWeb supports selecting an arbitrary number of samples for alignment. Configuration of the alignments is entered once through the user interface and applied consistently across all the jobs that will be started. (TIF) [file pone.0069666.s002.tif]

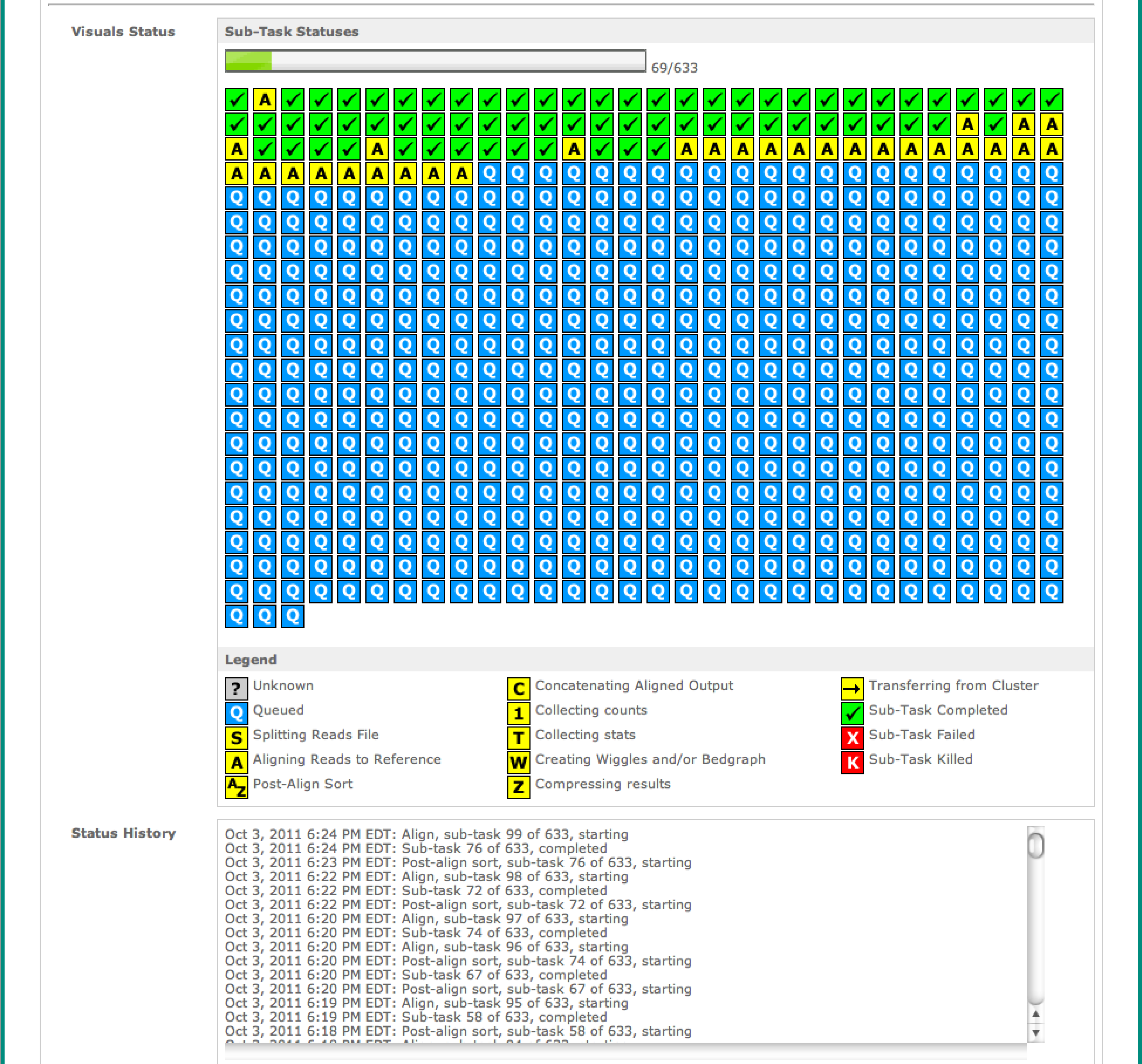

Supplement: Figure S3 — Visual status for alignment running on compute grid. The figure shows the visual status for an alignment in progress against a large sample (30 GB compressed reads were split into more than 600 chunks and were scheduled for alignment). GobyWeb aligns and sorts each chunk, then concatenates the sorted alignments pieces to yield a completely sorted alignment. Alignments are post-processed to derive base level histograms as well as statistics such as number of aligned reads and number of sequence variations at each cycle. (TIF) [file pone.0069666.s003.tif]

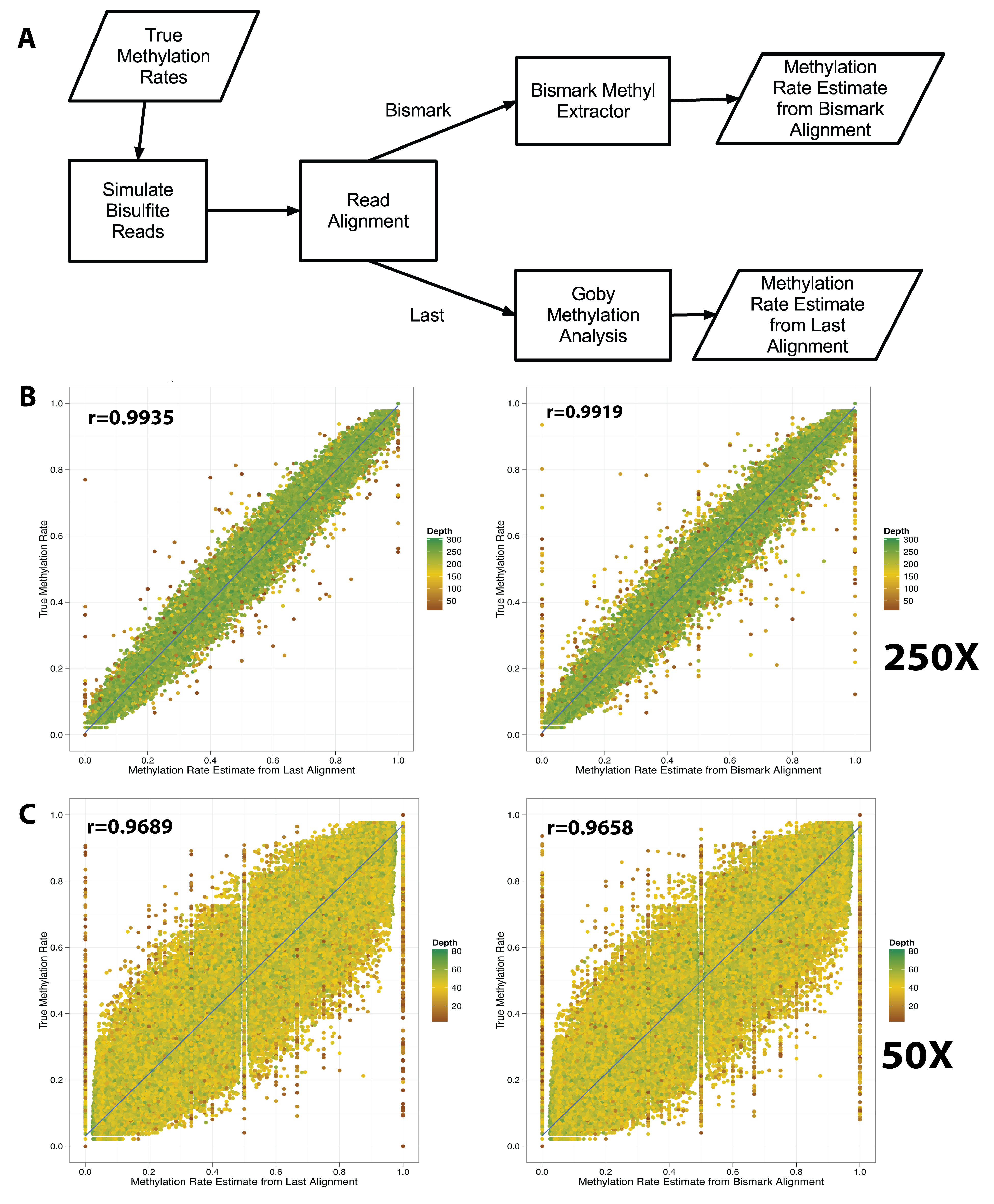

Supplement: Figure S4 — Comparison between estimates of methylation rates produced with Bismark and Last/Goby. GobyWeb can align bisulfite converted reads with either the Bismark or the Last aligner. Furthermore, alignments of bisulfite-converted reads can be processed to estimate methylation rates with either Goby or a simple script that post-processes the Bismark result files. Here, (A) we simulated reads from a uniform distribution of methylation rates over a 5 MB region of the human genome, at 50X or 250X average coverage and compare the estimate of methylation with the methylation estimate produced by each analysis method. We find (B) that both methods yield comparable agreement with true methylation rates and correlate well with each other when average coverage >50X (data simulated for a target of 50X coverage includes regions of the genome where actual coverage is lower than 50X, these sites tend to have larger disagreement with true methylation). (TIFF) [file pone.0069666.s004.tiff]
