# Supplementary material for: GobyWeb: Simplified Management and Analysis of Gene Expression and DNA Methylation Sequencing Data
Source: PLoS One. 2013 Jul 23;8(7):e69666. doi: 10.1371/journal.pone.0069666 (PMC3720652; doi:10.1371/journal.pone.0069666)
Supplement: File S1 — Supplemental Description and Figures S1-S4. (PDF) [file pone.0069666.s005.pdf]

Supporting Information for:

GobyWeb: simplified management and analysis of gene expression and DNA methylation sequencing data

Kevin C. Dorff[1], Nyasha Chambwe[2,3], Zachary Zeno[1], Manuele Simi[1], Rita Shaknovich[4], Fabien Campagne[1,2]*

[1]The HRH Prince Alwaleed Bin Talal Bin Abdulaziz Alsaud Institute for Computational Biomedicine.[2]Department of Physiology and Biophysics, [3]Tri-Institutional Training Program in Computational Biology and Medicine, [4]Department of Pathology and Department of Medicine; The Weill Cornell Medical College, New York, NY, USA.

* To whom correspondence should be addressed.

Description

In the following description, we will refer to biologists who use GobyWeb for data analysis as end-users. We will also refer to users who install and maintain an instance of GobyWeb on a local computing infrastructure as administrators.

Typical session. During a typical analysis project, a GobyWeb end-user often performs the following steps:

1. Imports data into GobyWeb (Actions->Upload menu)
2. Inspects uploaded data (pre-alignment quality control)
3. Aligns samples against a reference genome
4. Inspects alignment results (post-alignment quality control)
5. Compares groups of samples (the type of analysis varies with the kind of samples being compared)
6. View analysis results in web-browser or download results for processing with other tools
7. Package alignments for archival or to process alignments with custom scripts on a local machine
8. Share results or data with other users of GobyWeb, granting or obtaining access.

User registration. The Account menu makes it possible for new users to register with an instance of GobyWeb. Registering requires an invitation code, a username, a password and an email. The invitation code restricts registrations to those users who are invited to use the system. It is possible to disable the invitation code or print it on the registration page to entirely open the system. Registration makes it possible for the application to associate datasets to an owner, and formalize the concept of data sharing. Emails are collected to make it possible for the system to send notifications to end-users when analysis jobs are started or completed. Collecting emails also helps administrators notify end users in preparation of periodic system maintenance.

Uploading data. HTS reads can be uploaded to GobyWeb directly from the web browser (menu 1 in main manuscript Figure 1). The upload page uses a Java applet to make it possible to upload multi-gigabyte files. Files can be uploaded in compressed fasta, fastq or csfasta, fastq.gz.tar (produced by the Illumina CASAVA 1.8 pipeline) or in the Goby compact reads format. As an alternative to uploading via the web browser, GobyWeb supports two methods for uploading datasets from the command line. The first method is designed for sequencing facility staff members to transfer datasets directly to a GobyWeb user account. The second method makes it possible for individual users to upload data to their account from the command line, and is useful when end-users access the user interface via a network connection that cannot transfer large amounts of data (e.g., using a home network to upload and start an analysis when raw data resides within the institutional firewall).

Meta-data. The upload page makes it possible to describe meta-data about the read datasets. This option is available irrespective of the manner in which the data were uploaded. The following meta-data can be described: the technology used to sequence the reads (Illumina, SOLID, Helicos, Roche), the species/organism and the tissue whose biological material was assayed, a textual description of the protocol of the experiment, as well as whether the protocol used for library preparation preserved the strand. Some of these options will affect how GobyWeb runs alignments or conduct analyses and therefore should match the nature of the data uploaded.

Samples. GobyWeb defines the concept of a sample as the set of reads sequenced from the same biological or clinical material, for the purpose of analysis each sample should be considered homogeneous and independent of other samples. The check-box at the top of the upload page triggers the creation of independent samples when multiple files are uploaded, or the concatenation of the reads into a single sample. Samples whose reads are derived from DNA that has been bisulfite treated (i.e., Methyl-Seq or RRBS) can be marked as such. When files with the extension .fastq.gz.tar are uploaded, these files are assumed to have been produced by the CASVA 1.8 pipeline. In this case, the reads in each tar file (set of fastq.gz files) are concatenated to produce one sample. Sample upload also accepts a tab separated value file (.tsv extension) with a set of samples that can describe more custom read pairing and concatenation needs. The format of the TSV file is described online.

Data Sharing. Users can indicate that other end-users be given access to a dataset they own. End-users can share data at upload time, or at any time after upload by editing the dataset and adjusting the list of users that the item is shared with. Only owners of a data item can adjust sharing for this data item. When user A shares data with user B, the data appears to B as owned by user A. Clicking on a username in a list of data items provides information about the owner, including an email address. Users of GobyWeb can therefore communicate by email to request that another set of users be given access to a dataset by the owner.

Pre-alignment quality control. After samples are created, GobyWeb will start background analysis tasks that inspect the read files and collect quality control statistics. While this process is ongoing, the samples appear to the end user in the list of uploaded samples, but are marked as “not ready to align”. After the process completes, samples appear “ready to align” and can be selected to view collected statistics, or start alignments.

Aligning reads. End-users can align samples against a reference sequence. Users can either align a single sample by clicking on the Align link at the bottom of the sample page, or select the Align multiple samples option in the Actions menu. The alignment configuration page is shown in Figure 2. End-users can select one of several aligners: bwa (recommended for DNA-Seq), STAR or GSNAP (recommended for mRNA-Seq, GSNAP also supports bisulfite treated reads), last (recommended for smallRNA-Seq or when aligning to a reference different than that of the reads, also supports bisulte treated reads), or Bismark (for bisulfite converted reads). The subset of samples matching a specified organism is shown in a user interface component that supports interactive pattern matching (the list of samples is updated after every key-stroke with the set of samples that match the typed filter).

Alignments. Upon saving an alignment job, GobyWeb schedules the execution of the alignment in parallel on a compute grid. The end-user can obtain the status of submitted jobs in the “Browse>Running / completed alignments” menu item. Each individual alignment job also provides the status of its parallel computation on the grid (Figure 3). Upon completion, alignments can be downloaded in the Goby format, or in BAM format, either individually, or packaged as a ZIP file (archives can be produced with the “Action>Prepare alignments for download” menu item). Alignments are also post-processed to yield base level histograms in the Goby Count format.

Group comparisons. GobyWeb provides a multi-step software wizard to help end-users configure analyses that compare groups of samples. The wizards proceed in the following steps:

1. Define information about the group comparison analysis (e.g., number of groups to be compared, organism of the samples to be compared, description of the comparison analysis, type of analysis to perform, output format).

2. Select samples for the first group and name the group, repeat step 2 for each group defined in step 1.
3. Specify which pairs of groups need to be compared. Zero or more comparison pairs can be specified at this step. For instance, when comparing four groups (A, B, C, D), three groups may need to be compared to a reference group (i.e., A/B, A/C, A/D), or groups may be compared two by two such as (A/B, B/C, C/D).
4. Review the association between groups and samples, the pairs under comparison, and submit the analysis for execution.
5. Review results in table view, filter and download subset of tables, alternatively, download entire table in tab delimited or Variant Call format.

The following types of analyses are currently supported:

- Differential expression for gene, exon or CNV regions. Count of reads that overlap a region are compared across groups and statistics of differential expression evaluated.
- Differences in allelic expression (RNA-Seq). Heterozygote sites can be tested for difference in allelic ratios between groups. This analysis identifies heterozygous sites where the proportion of reference allele differs significantly between groups.
- Methylation rate analysis for individual bases or annotated regions. Alignments from bisulfite-converted samples can be used to estimate methylation rates at the cytosines sites of a genome and identify cytosine bases whose methylation rate differ significantly across groups.
- Calling genotypes. Genotype calls at all sites with at least 10x coverage can be generated. TSV and VCF files can be generated.
- Calling sites where alleles associate with groups. Genomic sites are identified where the count of alleles (expressed in number of samples with the allele in the group) differ across groups.

Exportable file formats. Intermediary and final results can be exported from GobyWeb to facilitate visualization or enable additional custom analyses.

Alignments and derived information can be packaged as Zip files and downloaded (point 7-8 in main manuscript Figure 1). Alignment can be downloaded either in the Goby format, or in BAM format (when the alignment was generated with the BAM output options shown in Figure 2). Both formats can be visualized with IGV to inspect specific regions of the genome and view mapping of individual reads.

Histograms. Goby base level histograms store the number of reads that cover a reference sequence, at every base of the reference, and can be downloaded and viewed as coverage tracks with IGV. Histograms are also produced in Wiggle or bedgraph format for visualization on the UCSC genome browser, or to process with bed-tools [1]. The Goby histogram format is recommended for viewing coverage in IGV because such files are typically 5 times smaller than equivalent bedgraph files.

Variant Calling Format. Genotypes, results of comparison of allele-specific expression, or differences in methylation rates are exportable either as tab-delimited files (from the GobyWeb table viewer) or in the Variant Calling Format (VCF 4.1) [2]. VCF files produced by GobyWeb are annotated with respect to which gene overlaps the genomic site described, the RefSNP (rs) number of the variation, and the expected effect of the variation (data obtained when available from Ensembl and biomart for the human reference genomes). VCF files are indexed and end-users can download both the VCF file and its index. VCF files produced by GobyWeb are compatible with IGV. An IGV extension recognizes the methylation rate format produced by GobyWeb and renders methylation rates as color gradients in a VCF track.

Supporting References

1. Quinlan AR, Hall IM (2010) BEDTools: a flexible suite of utilities for comparing genomic features. Bioinformatics 26: 841-842.
2. Danecek P, Auton A, Abecasis G, Albers CA, Banks E, et al. (2011) The variant call format and VCFtools. Bioinformatics 27: 2156-2158.

Supporting Figures

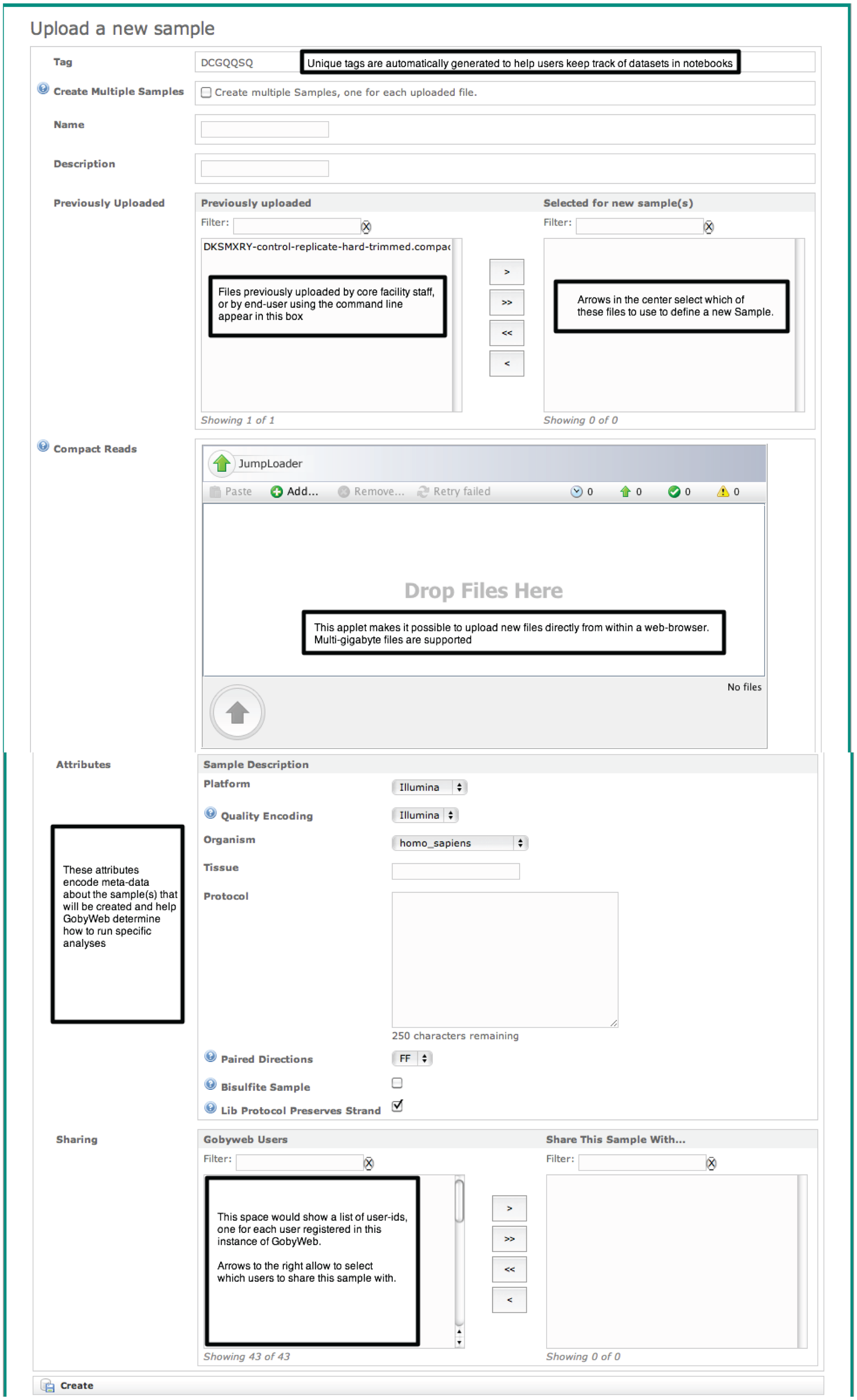

Figure S1. Uploading reads into GobyWeb to create a new Sample. Read files can be uploaded in a variety of file formats. When the checkbox "Create Multiple Samples" is not selected, individual files are concatenated to yield a single independent biological sample. When the box is not checked, multiple samples are created and associated with the meta-data described on the form.

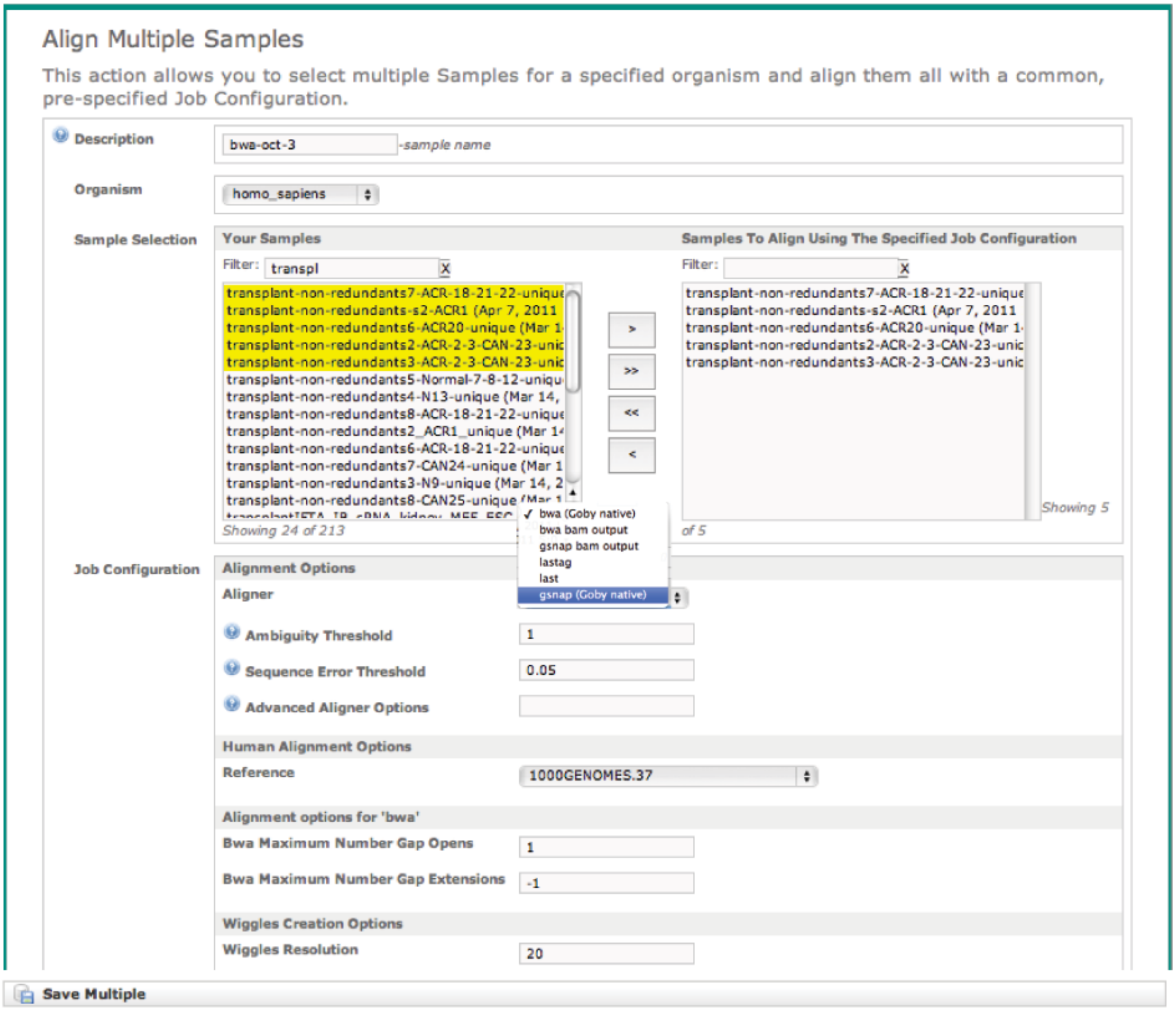

Figure S2. Consistent alignment of multiple samples. GobyWeb supports selecting an arbitrary number of samples for alignment. Configuration of the alignments is entered once through the user interface and applied consistently across all the jobs that will be started.

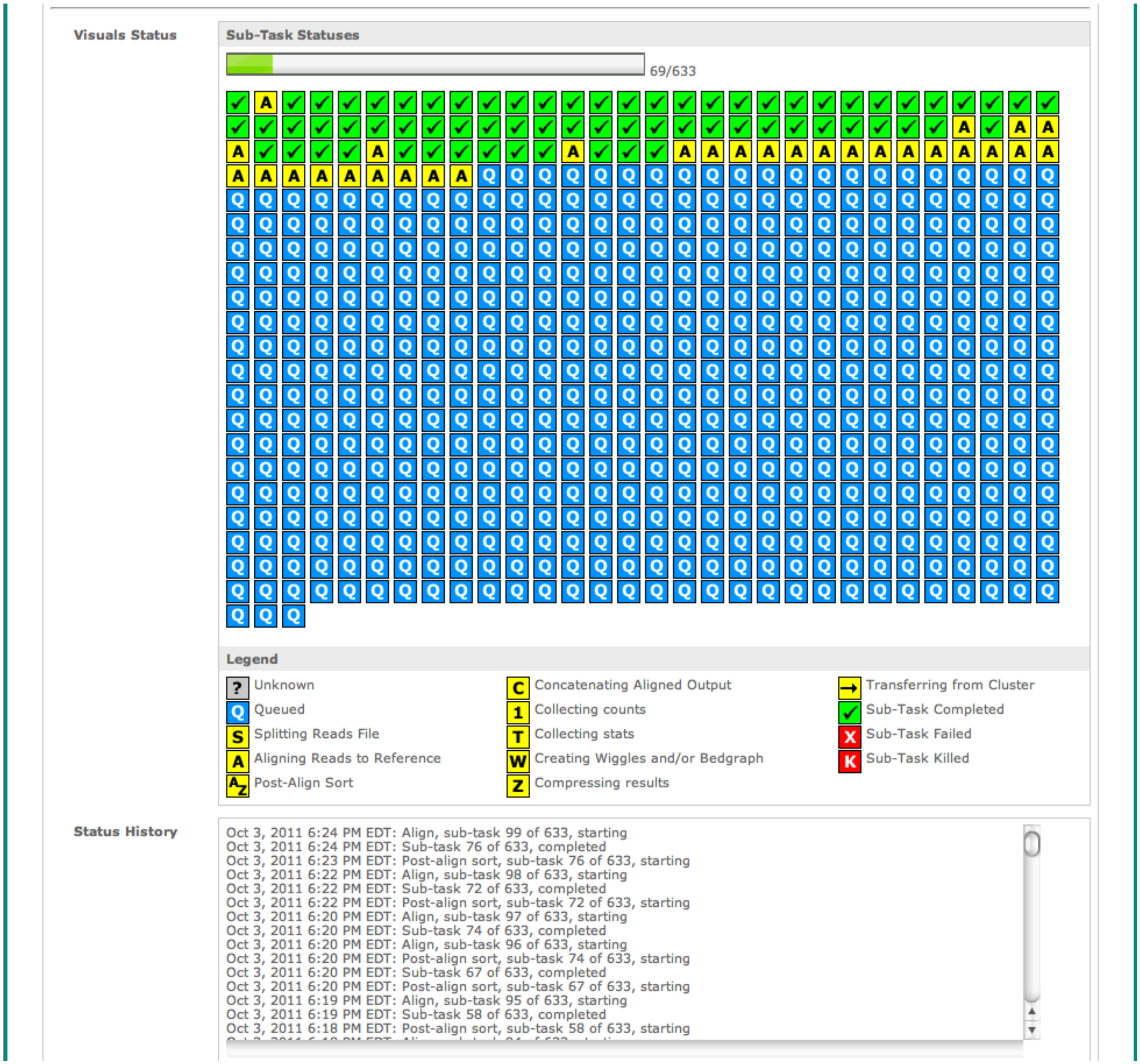

Figure S3. Visual status for alignment running on compute grid. The figure shows the visual status for an alignment in progress against a large sample (30GB compressed reads were split into more than 600 chunks and were scheduled for alignment). GobyWeb aligns and sorts each chunk, then concatenates the sorted alignments pieces to yield a completely sorted alignment. Alignments are post-processed to derive base level histograms as well as statistics such as number of aligned reads and number of sequence variations at each cycle.

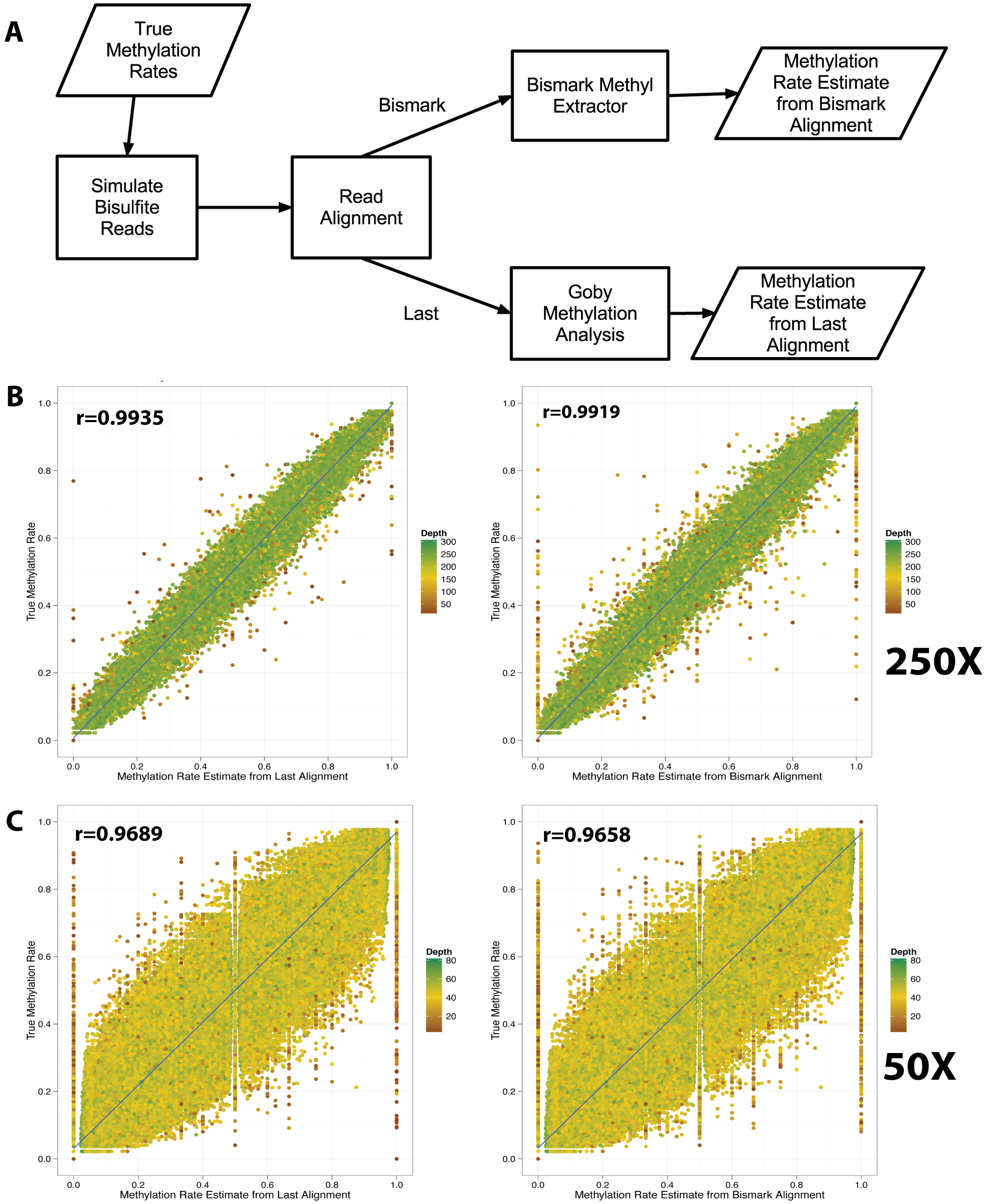

Figure S4. Comparison between estimates of methylation rates produced with Bismark and Last/Goby. GobyWeb can align bisulfite converted reads with either the Bismark or the Last aligner. Furthermore, alignments of bisulfite-converted reads can be processed to estimate methylation rates with either Goby or a simple script that post-processes the Bismark result files. Here, (A) we simulated reads from a uniform distribution of methylation rates over a 5MB region of the human genome, at 50X or 250X average coverage and compare the estimate of methylation with the methylation estimate produced by each analysis method. We find (B) that both methods yield comparable agreement with true methylation rates and correlate well with each other when average coverage >50X (data simulated for a target of 50X coverage includes regions of the genome where actual coverage is lower than 50X, these sites tend to have larger disagreement with true methylation).
